# Vitamin D supplementation in the first 2 years and autism spectrum traits at 6–8 years – a randomized clinical trial

**DOI:** 10.1111/jcpp.70110

**Published:** 2026-01-05

**Authors:** Samuel Sandboge, Vilja Seppälä, Sakari Lintula, Elisa Holmlund‐Suila, Helena Hauta‐alus, Eero Kajantie, Outi Mäkitie, Sture Andersson, Katri Räikkönen, Kati Heinonen

**Affiliations:** ^1^ Psychology/Welfare Sciences, Faculty of Social Sciences University of Tampere Tampere Finland; ^2^ Population Health Unit Finnish Institute for Health and Welfare Helsinki and Oulu Finland; ^3^ Department of Psychology, Faculty of Medicine University of Helsinki Helsinki Finland; ^4^ HUS Helsinki University Hospital and University of Helsinki Helsinki Finland; ^5^ Children's Hospital, Pediatric Research Center University of Helsinki and Helsinki University Hospital Helsinki Finland; ^6^ Research Program for Clinical and Molecular Metabolism (CAMM), Faculty of Medicine University of Helsinki Helsinki Finland; ^7^ Clinical Medicine Research Unit University of Oulu Oulu Finland; ^8^ Folkhälsan Institute of Genetics Helsinki Finland; ^9^ Department of Clinical and Molecular Medicine Norwegian University of Science and Technology Trondheim Norway; ^10^ Department of Molecular Medicine and Surgery, Karolinska Institutet, and Clinical Genetics Karolinska University Hospital Stockholm Sweden; ^11^ Department of Obstetrics and Gynaecology Helsinki University Hospital and University of Helsinki Helsinki Finland

**Keywords:** Vitamin D, neurodevelopment, randomized clinical trial, autism spectrum disorder

## Abstract

**Background:**

Early life vitamin D levels may be associated with autism spectrum disorder (ASD) and related traits, but causality is unknown. We examine whether higher‐than‐standard vitamin D_3_ supplementation during the first 2 years, as well as higher pregnancy and childhood 25‐hydroxyvitamin D (25(OH)D) levels and their trajectories, are associated with lower ASD trait scores at ages 6–8 years in a non‐clinical cohort.

**Methods:**

This secondary analysis of the double‐blind randomized clinical trial vitamin D intervention in infants (VIDI) comprised 366 Finnish children aged 6–8 years, 177 of whom were randomized to receive 400‐IU and 189 to receive 1,200‐IU daily oral vitamin D_3_ supplementation between ages 2 weeks and 2 years. ASD‐related traits were assessed at mean age 7.2 years (SD 0.4) using the parent‐reported Autism Spectrum Screening Questionnaire (ASSQ). Predictor variables were supplementation group, 25(OH)D concentrations measured during pregnancy and at ages 1 and 2 years, as well as 25(OH)D trajectories (high vs. low) derived from these time points.

**Results:**

None of the predictor variables of interest were associated with the outcome in the full sample. After sex stratification, among boys, 25(OH)D concentrations at 1 and 2 years were inversely associated with ASSQ scores (mean difference −0.2 of normalized SD score (95% CI −0.3 to −0.1, *p* = .003) and −0.2 (95% CI −0.3 to −0.05, *p* = .01) per 10 ng/mL 25(OH)D) after adjustment for age, breastfeeding, parental education, maternal depressive symptoms, and season of 25(OH)D assessment as was belonging to the higher 25(OH)D trajectory, −0.45 SD (95% CI −0.79 to −0.10, *p* = .01).

**Conclusions:**

We found no indication that higher‐than‐normal vitamin D_3_ supplementation between ages 0 and 2 years decreases ASD‐related trait scores at ages 6–8 years. Sex‐stratified analysis suggested an inverse association, among boys, between early life 25(OH)D concentrations and ASD‐related traits, warranting further studies on potential causal direction and sex specificity of associations.

## Introduction

Autism spectrum disorder (ASD) is a complex early appearing neurodevelopmental disability characterized by stereotypical and repetitive behaviors, difficulties in social interactions, and communication impairments. The global prevalence of ASD is estimated to be 0.6–0.7% and the male–female ratio is 4.3:1 (Salari et al., 2022; Talantseva et al., 2023). However, behavioral traits of ASD are more common and a dimensional conceptualization, rather than a strictly categorical diagnostic approach, is being suggested (Abu‐Akel, Allison, Baron‐Cohen, & Heinke, 2019). The etiology of ASD is multifactorial, and several potential genetic and environmental risk factors have been identified (Fang et al., 2023; Masini et al., 2020). Vitamin D—usually measured as blood 25‐hydroxyvitamin D (25(OH)D) concentration—is a pleiotropic steroid hormone, which, in addition to its role in bone health, is also involved in neurodevelopment and thus suggested as one of the etiological factors for ASD and related behavioral traits (Cannell, 2008).

While details regarding a potential causal link between prenatal vitamin D levels and ASD remain unclear, several potential underlying mechanisms for such an association have been suggested, including the role of vitamin D in neuronal differentiation, regulation of synaptic plasticity, reduction of oxidative burden, and proliferation and apoptosis (Bivona, Gambino, Iacolino, & Ciaccio, 2019). A causal link is also supported by animal studies, as reported by, e.g., Ali et al. (2019) in a rat model study linking developmental vitamin D deficiency with autism‐related phenotypes. In regard to observational studies in humans, a meta‐analysis of prospective studies demonstrated that lower maternal pregnancy or neonatal 25(OH)D concentrations were linked to a higher likelihood of later ASD (Wang, Ding, & Wang, 2020). A Finnish register study replicated an inverse association between maternal pregnancy 25(OH)D concentrations and offspring ASD (Sourander et al., 2021). Studies on the association between prenatal vitamin D levels and ASD‐related traits – rather than ASD diagnosis – are more sparse and inconsistent; two previous prospective cohort studies demonstrated inverse associations between maternal gestational 25(OH)D concentrations and later ASD‐related traits (Madley‐Dowd et al., 2022; Vinkhuyzen et al., 2018), whereas another study's findings were mainly non‐significant (Whitehouse et al., 2013). Contrasting the hypothesis of causality, some studies instead suggest that a genetic overlap between vitamin D levels and ASD may underlie the observed associations (Fang et al., 2023; Yu, Xu, Chen, & Ke, 2023).

. While observational studies, as summarized in, e.g., a meta‐analysis of 24 observational case–control studies (Wang et al., 2020), report significantly lower childhood and adolescent 25(OH)D concentrations among individuals with ASD compared with controls, other studies suggest a modulating effect of vitamin D supplementation. For example, a recent review and meta‐analysis by Zhang et al. (2023) suggested a beneficial effect of vitamin D supplementation on stereotypical behaviors in children with ASD. The plausibility of a postnatal intervention window is also supported by animal studies. For example, Du, Zhao, Duan, and Li (2017) reported behavioral improvements in rats with valproic acid‐induced ASD‐like behaviors after early supplementation with vitamin D.

In summary, previous studies have primarily focused on two questions: (1) The potential causal impact of prenatal vitamin D levels on ASD diagnosis and ASD‐related traits, and (2) Vitamin D as a symptom modulator among children with ASD diagnosis. Given the suggested impact of vitamin D on the postnatal neurodevelopmental period of plasticity, there is, however, a lack of studies exploring potential critical or sensitive periods. Additionally, to our knowledge, there are no observational studies evaluating the potential impact of early childhood vitamin D on ASD‐related traits in non‐clinical populations.

In a previous Vitamin D Intervention in Infants (VIDI) randomized clinical trial publication, we demonstrated that children who received a higher‐than‐normal (1200‐IU) daily vitamin D_3_ supplementation between ages 2 weeks and 2 years had lower prevalence of internalizing psychiatric problems at ages 6–8 years, compared with those who received standard recommended supplementation dosage (400‐IU) (Sandboge et al., 2023). Internalizing problems are especially common in children with ASD (Dellapiazza, Audras‐Torrent, Michelon, & Baghdadli, 2021; Guerrera et al., 2019) and given our findings on the potentially positive impact of vitamin D_3_ supplementation during early neurodevelopment, as well as previous findings on ASD and vitamin D, the primary aim of the current study was to assess whether a higher‐than‐normal vitamin D supplementation could give similar benefits regarding ASD‐related traits. Our secondary aim was to fill the knowledge gap of potential sensitive periods of vitamin D levels in relation to ASD traits. We explored the associations between early life vitamin D levels and trajectories and ASD‐related traits, using 25(OH)D serum samples collected from the mother during pregnancy, and from the child at ages 1 and 2 years. Finally, due to the unequal sex ratio in ASD, we aimed to test whether associations between ASD traits and vitamin D supplementation vary by sex.

## Methods

This study is a randomized clinical trial secondary analysis. Informed consent forms were collected from parents at recruitment, and from parents and children at the 6–8‐year follow‐up. The study was approved by the ethics committee at the Hospital District of Helsinki and Uusimaa, follows the Consolidated Standards of Reporting Trials (CONSORT) guidelines, and is registered with ClinicalTrials.gov (NCT01723852 [VIDI], and NCT04302987 [VIDI2]).

### Study design and participants

**Figure 1:**
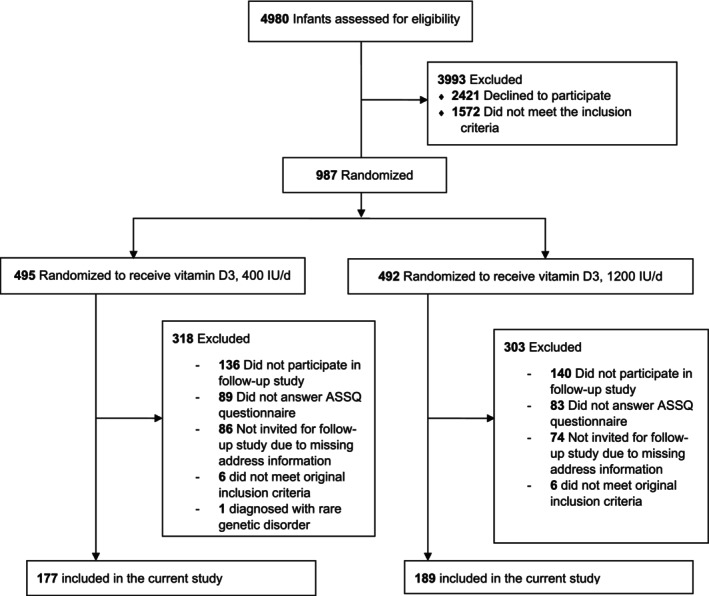
Flowchart of study enrollment, allocation, and follow‐up

The VIDI study, a randomized, double‐blind clinical trial, has been described elsewhere in detail (Hauta‐Alus et al., 2021; Helve et al., 2017; Rosendahl et al., 2018). The original study population comprised 987 families (492 girls and 495 boys, with mothers of Northern European origin), 12 of whom were subsequently excluded due to not fulfilling inclusion criteria (as described in Appendix S1 and Figure 1). Recruitment took place between January 14, 2013, and June 9, 2014, at the Kätilöopisto Maternity Hospital, Helsinki, Finland, 60° North Latitude. Infants were randomized to receive either 400‐IU (10 μg) (*n* = 489) or 1,200‐IU (30 μg) (*n* = 486) daily oral vitamin D_3_ supplementation between ages 2 weeks and 2 years. Orion Pharmaceuticals provided the supplements; participants from both groups received five drops daily. A Helsinki University Hospital pharmacist, unrelated to the study, performed randomization in blocks of 50. Investigators and participating families were blinded to group assignment. Parents were informed of group membership at the conclusion of the original 2‐year intervention. Information on parental lifestyle, health, and demographics was collected using standardized questionnaires. Hospital records provided data on gestation, delivery, and child demographics. All maternal 25(OH)D concentrations were measured from serum samples collected during routine maternity clinic visits at gestational weeks 6–27 (mean = 11.3, SD = 1.9) and stored in the Finnish Maternity Cohort serum bank, organized by the National Institute for Health and Welfare.

### Follow‐up at age 6–8‐years

After the original 2‐year VIDI study's conclusion, all remaining participating families with available home address information (*n* = 817) were invited to the VIDI2 follow‐up study at ages 6–8 years. Of the 546 families who participated in VIDI2, 456 completed online questionnaires on psychiatric and cognitive outcomes between September 2020 and May 2021. The current study population comprises 366 children (177 (36%) from the original 400‐IU supplementation group and 189 children (39%) from the 1,200‐IU group) whose parents completed the Autism Spectrum Screening Questionnaire (ASSQ) (Ehlers, Gillberg, & Wing, 1999). Compared with those who were lost to follow‐up, the participants of the current study had somewhat more favorable baseline characteristics (Appendix S1 and Table S1) (longer breastfeeding duration, higher parental education, and less maternal smoking). Additionally, a higher proportion of participants were born during summer and autumn, compared with nonparticipants.

### Biochemical analysis

25(OH)D was measured from maternal serum during pregnancy, and serum collected from study participants at ages 1 and 2 years. Samples were analyzed using a fully automated IDS‐iSYS immunoassay system with chemiluminescence detection (Immunodiagnostics System Ltd., Bolton UK). Biochemical analyses have been described elsewhere in detail (Helve et al., 2017; Rosendahl et al., 2018). We used 30 ng/mL as a cutoff point for lower 25(OH)D concentrations (Holick et al., 2011) in accordance with our previous publication (Sandboge et al., 2023).

### Outcome measures

ASD‐related traits were assessed at age 6–8 years using the parent‐reported ASSQ which includes 27 items rated on a 3‐point scale (0 = normal; 1 = some abnormality; 2 = definite abnormality) (Ehlers et al., 1999). ASSQ measures communication problems, restricted and repetitive behavior, social interaction, motor clumsiness and other ASD‐associated symptoms in clinical and non‐clinical samples. Higher scores indicate more ASD‐related traits. ASSQ has been validated for use in the Finnish population, for medium‐ to high‐risk samples and in general population settings (Mattila et al., 2012) and has good psychometric properties (Ehlers et al., 1999). In the current study, Cronbach's *α* for the scale's internal consistency was 0.84. We square‐root‐transformed sum scores, and then standardized them (mean 0, standard deviation 1), and used these *z*‐scores as the outcome measure. Children with ASSQ scores of 17 points and above, i.e., the suggested diagnostic cutoff point for ASD (Posserud, Lundervold, & Gillberg, 2009), were identified but analysis was limited to the sum score, given very low prevalence figures (*n* = 5).

### Covariates

Potential covariates were evaluated based upon previously described associations with vitamin D status and/or neurodevelopment, i.e., child's sex (McEwen & Milner, 2017), maternal age (Tearne, 2015), length of gestation (Pierrat et al., 2017), season of birth (Asano et al., 2016; Bai et al., 2018), duration of breastfeeding (Darmawikarta et al., 2016), age at assessment (Solmi et al., 2022), parental education (Bradley & Corwyn, 2002), parental single status at follow‐up (Daryanani, Hamilton, Abramson, & Alloy, 2016), smoking (Polańska, Jurewicz, & Hanke, 2015), maternal anxiety (Ghirardi et al., 2021), and maternal depressive symptoms (Tuovinen et al., 2018). Sex, breastfeeding duration (months), maternal depressive symptoms (Center for Epidemiological Studies Depression Scale (CES‐D), questionnaire received after delivery), parental education (low = less than a bachelor's degree, high = bachelor's degree or above), and season of laboratory test (categorized into four seasons) were all associated with ASSQ scores (*p* < .05) (Table S2) and therefore included in the analyses. All covariates are described in more detail in Appendix S1.

### Statistical analysis

This is a secondary analysis of the VIDI randomized clinical trial. We followed a per‐protocol design, with participant data analyzed according to original intervention group assignment, regardless of intervention compliance. Follow‐up characteristics between intervention groups were compared using two‐tailed independent samples *t*‐tests and Pearson *χ* ^2^‐tests. Differences in ASSQ scores between groups were assessed using linear regression analysis. The crude model presents unadjusted associations whereas the adjusted model includes sex, breastfeeding duration, maternal depressive symptoms during the early postpartum period, and parental education as covariates. In all regression analyses, heteroskedasticity consistent standard errors and respective confidence intervals were used. Setting *α* at 0.05, we had a power of 0.80 to exclude or confirm effect sizes greater than 0.30 in mean difference (MD) when comparing intervention groups. Missing covariate data were imputed, as described in Appendix S1. The magnitude, directions, and significance levels of associations in the full model were similar to those in a reduced model, excluding individuals with missing data (Appendix S1). To test for potential attrition bias, we performed inverse probability weighting estimation. Associations between the main predictors of interest and ASSQ score were tested using the same covariates as in the original models (Appendix S1 and Table S3). False discovery rate (FDR) correction using the Benjamini–Hochberg procedure was applied to the pooled and sex‐stratified analyses separately to account for multiple testing.

Linear regression analysis was used to examine the associations between 25(OH)D concentrations measured during pregnancy and at ages 1 and 2 years, and ASSQ scores. Adjustments include season of lab measurement, in addition to those described above, to account for associations between 25(OH)D and season (Table S4). Analyses were run with 25(OH)D as a continuous variable and dichotomized at 30 ng/mL.

In a post hoc exploratory analysis, we employed latent profile analysis (LPA) to identify potential distinct 25(OH)D trajectories from pregnancy through childhood up to age 2 years (described in detail in Appendix S1 and Figure S1). An a priori assumption of two separate trajectories – one with systematically higher mean 25(OH)D concentrations at all measurement points compared with the other – was supported by data analysis. The dichotomous trajectory variable was entered into regression models, replacing the supplementation group variable.

Interaction analysis, described in Appendix S1, indicated interaction between sex and 1‐ and 2‐year 25(OH)D concentrations (*p* = .01 and *p* = .05, respectively) in relation to ASSQ scores. Hence, all analyses are also presented separately for boys and girls. We additionally tested for potential interactions between maternal 25(OH)D levels and supplementation group status and 25(OH)D concentrations at ages 1 and 2 years, respectively, and given lack of interaction (*p* = .24, *p* = .18, and *p* = .16, respectively), analyses were not stratified by maternal 25(OH)D levels. Lastly, we conducted additional post hoc analyses to examine the role of childhood internalizing psychiatric problems in the association between 25(OH)D status and ASD‐related traits. However, because of the potential for overadjustment due to collider bias, as described in Appendix S1, these analyses are reported only as supplemental information (see Appendix S1 and Tables S5 and S6).

Statistical analyses were performed using SPSS (IBM SPSS Statistics for Windows, version 28) and R version 4.3.0 (‘R: A Language and Environment for Statistical Computing’, 2024). LPA was performed using the “mclust” package version 5 in R (Scrucca, Fraley, Murphy, & Raftery, 2023).

## Results

The study population included 366 children: 177 (85, 48% girls) in the 400‐IU and 189 (91, 48% girls) in the 1,200‐IU supplementation group with an average age of 7.1 years (SD = 0.4). Table 1 presents baseline characteristics. Tables S1 and S7 compare baseline characteristics by participation vs. non‐participation and by sex, respectively. Children in the 1,200‐IU supplementation group had significantly higher 25(OH)D concentrations at ages 1 and 2 years; MD 13.2 (11.0–15.3; *p* < .001), 12.5 (10.6–14.4; *p* < .001) ng/mL, respectively (Table 2). The prevalence ASSQ scores above the cutoff point was 1.1% (2/177) in the 400‐IU supplementation group and 1.6% (3/189) in the 1,200‐IU supplementation group (*p* = .71).

**Table 1:** Baseline characteristics by intervention groups

Characteristic	400‐IU vitamin D group (*n* = 177)		1,200‐IU vitamin D group (*n* = 189)		MD (95% CI)	*p* Value^a^
Child						
Female sex, no. (%)	85 (48)	177	91 (48)	189		.98
Gestational length, mean (SD), d	280.4 (7.7)	177	281.2 (7.4)	189	0.8 (−0.8; 2.3)	.33
Season of birth		177		189		
Winter, no. (%)	30 (16.9)		34 (18.0)			.79
Spring, no. (%)	68 (38.4)		64 (33.9)			.36
Summer, no. (%)	41 (26.6)		52 (27.5)			.34
Autumn, no. (%)	38 (21.5)		39 (20.6)			.85
Mother						
Age at delivery, mean (SD), y	31.6 (4.0)	174	31.4 (4.3)	189	−0.2 (−1.0; 0.7)	.73
Smoking at childbirth (yes), no. (%)	20 (11.4)	176	25 (13.2)	189		.59
Pregnancy 25(OH)D concentration						
Mean (SD), ng/mL	33.3 (9.3)	151	33.7 (7.9)	157	0.4 (−1.6; 2.3)	.70
< 30 ng/mL, no. (%)	56 (37.0)	151	46 (29.3)	157		.15
Educational level (high), no. (%)	125 (83.3)	150	130 (82.8)	157		.90
Depressive symptoms at childbirth, median (IQR), score^b^	11.0 (8.0–16.0)	164	10.0 (8.0–15.0)	169		.74
CES‐D score ≥16 no. (%)^c^	43 (26.2)	164	34 (20.1)	169		.19
Father						
Educational level (high), no. (%)	100 (67.6)	148	107 (69.9)	153		.75

SI conversion factor: To convert 25(OH)D to nmol/L, multiply by 2.496. 25(OH)D, serum 25‐hydroxyvitamin D; CES‐D, Center for Epidemiological Studies Depression Scale; d, days; IU, International Units; m, months; SD, standard deviations; y, years.

^a^ *p* values for proportions calculated using *χ* ^2^ test; *p* values for medians calculated using the Mann–Whitney *U* test; *p* values for means calculated using *T*‐test, two‐sided *p* values, equal variance not assumed.

^b^ Depressive symptoms assessed using the Center for Epidemiological Studies Depression Scale.

^c^ Scores of 16 or above indicate a risk of clinical depression.

**Table 2:** Follow‐up characteristics by intervention group

Characteristic	400‐IU vitamin D group (*n* = 177)		1,200‐IU vitamin D group (*n* = 189)		MD (95% CI)	*p* value^a^
Child						
At 12‐month follow‐up						
Length of breastfeeding, mean (SD), months	11.4 (5.8)	167	11.0 (5.4)	177	−0.4 (−1.6; 0.7)	.45
25(OH)D concentration						
Mean (SD), ng/mL	34.0 (8.1)	167	47.2 (11.7)	176	13.2 (11.0; 15.3)	<.001
< 30 ng/mL, No. (%)	58 (34.7)	167	5 (2.8)	176		<.001
At 24‐month follow‐up						
25(OH)D concentration						
Mean (SD), ng/mL	35.5 (8.0)	176	48.0 (10.1)	187	12.5 (10.6; 14.4)	<.001
< 30 ng/mL, No. (%)	50 (28.4)	176	6 (3.2)	187		<.001
At 6–8‐year follow‐up						
Age, mean (SD), y	7.2 (0.4)	177	7.2 (0.4)	189	−0.02 (−0.1; 0.1)	.72
ASSQ score, median (IQR)	2.0 (1.0–5.0)	177	2.0 (1.0–4.0)	189		.97
Belonging to low 25(OH)D trajectory group, no. (%)	105 (72.9)	144	41 (28.1)	146		<.001

ASSQ, Autism Spectrum Screening Questionnaire; CI, confidence interval; IQR, interquartile range; IU, International Units; MD, mean difference; SD, standard deviation.

^a^ *p* values for proportions calculated using *χ* ^2^ test; *p* values for medians calculated using the Mann–Whitney *U* test; *p* values for means calculated using *T*‐test, two‐sided *p* values, equal variance not assumed.

### Vitamin D supplementation during early childhood and ASD‐related traits at ages 6–8 years

We found no differences in ASSQ scores between supplementation groups. The mean difference in square‐root‐transformed standardized ASSQ score was −0.002 (95% CI −0.20 to 0.20, *p* = .99) after adjustments (Table 3). Associations remained non‐significant in the sex‐stratified analysis (Table 4).

**Table 3:** Association between predictor variables and ASD symptoms at age 6–8 years^a^

	*n*	*B* (95% CI)	*p* Value
Supplementation group, 1,200‐IU vs. 400‐IU			
Crude^b^	366	−0.002 (−0.21; 0.20)	.98
Adjusted^c^	366	−0.002 (−0.20; 0.20)	.98
25(OH)D trajectory group, high vs. low			
Crude	290	−0.24 (−0.46; −0.01)	.04
Adjusted	290	−0.22 (−0.44; −0.004)	.05
Maternal 25(OH)D levels during pregnancy			
Crude	308	−0.01 (−0.02; 0.01)	.31
Adjusted	308	−0.004 (−0.02; 0.01)	.54
Maternal 25(OH)D levels > vs. <30 ng/mL			
Crude	308	−0.01 (−0.25; 0.22)	.92
Adjusted	308	0.04 (−0.21; 0.28)	.77
Child's 1‐year 25(OH)D levels			
Crude	343	−0.01 (−0.02; 0.002)	.14
Adjusted	343	−0.01 (0.02; 0.003)	.16
Child's 1‐year 25(OH)D levels > vs. <30 ng/mL			
Crude	343	−0.10 (−0.36; 0.16)	.46
Adjusted	343	−0.05 (−0.30; 0.20)	.68
Child's 2‐year 25(OH)D levels			
Crude	363	−0.01 (−0.02; 0.001)	.09
Adjusted	363	−0.01 (−0.02; 0.002)	.12
Child's 2‐year 25(OH)D levels > vs. <30 ng/mL			
Crude	363	−0.30 (−0.59; −0.01)	.04
Adjusted	363	−0.26 (−0.53; 0.02)	.07

Abbreviations: IU, International Units; B, effect size; CI, confidence interval; ASSQ, Autism Spectrum Screening Questionnaire.

^a^ Autism spectrum disorder symptoms assessed using the Autism Spectrum Screening Questionnaire. Raw scores were square‐root‐transformed due to skewness and converted to *Z*‐scores (0 = mean, 1 = 1 SD). Effect sizes were calculated as mean differences between the 1,200 IU and 400 IU supplementation groups and latent profile analysis groups, as well as change in standardized square‐root‐transformed ASSQ score per ng/mL increase of 25(OH)D predictor variables.

^b^ Unadjusted model.

^c^ Adjusts for sex, age at assessment, breastfeeding duration, maternal education, paternal education, and maternal depressive symptoms at birth. The 25(OH)D predictor variable models additionally include adjustment for the season the lab test was collected. Missing values for maternal depressive symptoms (*n* = 33), season of 1 year lab test (*n* = 1), and breastfeeding duration (*n* = 1) were imputed.

**Table 4:** Association between predictor variables and ASD symptoms at age 6–8 years^a^, stratified by sex

	Boys			Girls		
	*B* (95% CI)	*p* Value	*n*	*B* (95% CI)	*p* Value	*n*
Supplementation group, 1,200‐IU vs. 400‐IU						
Crude^b^	0.02 (−0.28; 0.32)	.92	190	−0.02 (−0.29; 0.25)	.88	176
Adjusted^c^	0.02 (−0.28; 0.32)	.90	190	−0.01 (−0.27; 0.25)	.95	176
25(OH)D trajectory group, high vs. low						
Crude^b^	−0.41 (−0.75; −0.08)	.02	147	0.01 (−0.28; 0.30)	.95	143
Adjusted	−0.45 (−0.80; 0.10)	.01	147	0.004 (−0.27; 0.28)	.98	143
Maternal 25(OH)D concentration						
Crude	−0.01 (−0.02; 0.01)	.46	159	−0.01 (−0.03; 0.01)	.29	149
Adjusted	−0.004 (−0.02; 0.01)	.68	159	−0.01 (−0.02; 0.01)	.50	149
Maternal 25(OH)D levels > vs. <30 ng/mL						
Crude	0.08 (−0.27; 0.43)	.65	159	−0.07 (−0.37; 0.24)	.66	149
Adjusted	0.14 (−0.23; 0.52)	.44	159	−0.12 (−0.42; 0.19)	.45	149
1‐year 25(OH)D concentration						
Crude	−0.02 (−0.03; −0.004)	.02	176	0.004 (−0.01; 0.02)	.47	167
Adjusted	−0.02 (−0.03; −0.01)	.003	176	0.01 (−0.01; 0.02)	.42	167
Child's 1‐year 25(OH)D levels > vs. <30 ng/mL						
Crude	−0.31 (−0.71; 0.08)	.09	176	0.21 (−0.10; 0.52)	.26	167
Adjusted	−0.41 (−0.78; −0.03)	.03	176	0.31 (−0.04; 0.65)	.08	167
2‐year 25(OH)D concentration						
Crude	−0.01 (−0.03; 0.00)	.04	188	0.001 (−0.01; 0.01)	.81	175
Adjusted	−0.02 (−0.03; −0.005)	.01	188	0.004 (−0.01; 0.02)	.53	175
Child's 2‐year 25(OH)D levels > vs. <30 ng/mL						
Crude	−0.45 (−0.82; −0.09)	.02	188	0.03 (−0.39; 0.43)	.98	175
Adjusted	−0.53 (−0.89; −0.17)	.004	188	0.12 (−0.28; 0.52)	.54	175

ASSQ, Autism Spectrum Screening Questionnaire; *B*, effect size; CI, confidence interval; IU, International Units.

^a^ Autism spectrum disorder symptoms assessed using the Autism Spectrum Screening Questionnaire. Raw scores were square‐root‐transformed due to skewness and converted to *Z*‐scores (0 = mean, 1 = 1 *SD*). Effect sizes were calculated as mean differences between the 1,200 IU and 400 IU supplementation groups and latent profile analysis groups, as well as change in standardized square‐root‐transformed ASSQ score per ng/mL increase of 25(OH)D predictor variables.

^b^ Unadjusted model.

^c^ Adjusts for age at assessment, breastfeeding duration, maternal education, paternal education, and maternal depressive symptoms at birth. The 25(OH)D predictor variable models additionally include adjustment for the season the lab test was collected. Missing values for maternal depressive symptoms (*n* = 33), season of 1 year lab test (*n* = 1), and breastfeeding duration (*n* = 1) were imputed.

### Early life 25(OH)D concentrations and ASD‐related traits at ages 6–8 years

We found no statistically significant associations between continuous 25(OH)D concentrations (pregnancy, childhood 1‐ and 2‐year) and ASSQ scores (Table 3). Having a 25(OH)D concentration >30 ng/mL at age 2 years was, however, related to lower ASSQ scores in the crude model (*p* = .04) but was not significant after adjustment for covariates (*p* = .07).

In the sex‐stratified analysis, among boys, 25(OH)D concentrations at ages 1 and 2 years were inversely associated with ASSQ (*p* values <.01 after covariate adjustment, *p* values <.05 after further FDR‐correction for multiple testing). Maternal 25(OH)D concentrations were not associated with ASSQ in either sex, nor were 1‐ and 2‐year 25(OH)D concentrations in girls (Table 4).

### 25(OH)D trajectories and ASD‐related traits at ages 6–8 years

After adjustment for covariates, those belonging to the trajectory group with higher 25(OH)D concentrations at all time points had lower ASSQ scores at ages 6–8 years (*p* < .05) (Table 3). The association, however, did not survive FDR‐corrected (*p* = .25). In the sex‐stratified analysis (Table 4), boys in the higher 25(OH)D trajectory had a 0.45 SD (95% CI 0.10 to 0.80, *p* = .01; FDR‐corrected *p* = .03) lower ASSQ score after adjustments, corresponding to a difference in raw ASSQ score of −1.2 (95% CI −1.8 to −0.3). No statistically significant differences were seen for girls.

## Discussion

In this secondary analysis of the VIDI randomized clinical trial, we found no indication that three times higher‐than‐standard vitamin D_3_ supplementation during the first 2 years of life decreases ASD‐related traits at ages 6–8 years in a cohort of healthy term‐born children. Childhood 25(OH)D concentrations, however, were inversely associated with ASD trait scores, albeit only among boys. LPA identified two distinct 25(OH)D trajectories, one characterized by higher 25(OH)D concentrations throughout pregnancy and childhood, the other by lower concentrations. Boys with a lower 25(OH)D trajectory also had higher ASD trait scores, on average. Although results from previous case–control studies indicate that children and adolescents with ASD diagnosis have lower 25(OH)D levels compared with their peers, this is, to our knowledge, the first study that demonstrates an association with ASD‐related traits in a non‐clinical cohort.

Previous studies have implicated prenatal 25(OH)D levels as a potential etiological factor in the development of ASD and related behavioral traits. In our study, we found no association between maternal pregnancy 25(OH)D concentrations and ASD‐related traits at ages 6–8 years. Gestational vitamin D deficiency (25(OH)D < 10 ng/mL) has previously been linked to ASD‐related traits, as reported in a study of 4,229 mother–child pairs from the Dutch Generation R population‐based cohort study (Vinkhuyzen et al., 2018). Importantly though, the prevalence of maternal 25(OH)D levels <10 ng/mL was markedly higher in Generation R, compared with the VIDI study (16% (Sammallahti et al., 2021) vs. 0.2% in the original VIDI study population (*n* = 777)). A Danish randomized clinical trial, featuring 591 mother–child pairs from the general population demonstrated an inverse association between gestational vitamin D levels and autism traits at age 10 years but saw no benefit of high‐dose (2800‐IU/day) vitamin D supplementation during mid‐late pregnancy, compared with a lower (400‐IU/day) dosage (Aagaard et al., 2024). In a UK study featuring 7,689 births, some evidence was found for increased odds of autism‐associated traits in offspring of mothers with lower pregnancy 25(OH)D levels (Madley‐Dowd et al., 2022). In a study from the Australian Raine cohort, featuring 406 participants, no associations between maternal 25(OH)D concentrations and offspring autistic traits in early adulthood were found, with the exception that offspring of mothers with 25(OH)D concentrations <50 nmol/L had an increased risk of attention switching difficulties (Whitehouse et al., 2013).

Apart from our study and the ones listed above, previous research has focused on the association between vitamin D concentrations and ASD diagnosis, rather than on ASD‐related traits. Many of these studies are based on measurements collected during childhood, in some cases considering vitamin D levels as a modifiable risk factor for ASD symptom severity. A systematic review and meta‐analysis, featuring 20,580 participants from 34 studies, found that children and adolescents with ASD had significantly lower 25(OH)D concentrations, compared with controls and additionally that lower maternal or neonatal 25(OH)D concentrations were associated with an increased risk of ASD diagnosis (Wang et al., 2020). Previous randomized clinical trials, excluding the study by Aagard et al (2023), have focused on children with confirmed diagnosis. A recent review of 8 randomized controlled trials featuring children with ASD, 6 of which (comprising 176 children) were included in a meta‐analysis, found that vitamin D supplementation was associated with decreased stereotypical behaviors but had no other impact on core symptoms or coexisting conditions (Zhang et al., 2023) suggesting that supplementation may have a measurable, albeit limited, modulatory impact. In our study, only 5 children (1.4% of the participants, 2 from the 400‐IU group and 3 from the 1,200‐IU group) scored above ASSQ's suggested diagnostic cutoff point, limiting direct comparison to studies with diagnosed participants.

Our finding on the associations between early childhood 25(OH)D trajectories and levels and later ASD‐related traits was restricted to boys. Based on the information collected and analyzed in the current study, we are unable to assess whether the observed sex differences may have underlying biological reasons, or whether they may be explained by diagnostic challenges in finding traits associated with a female ASD‐phenotype, as discussed by, e.g., Kopp and Gillberg (2011). Given sex differences in ASD‐prevalence – 3–4:1 in population cohorts and 5–14:1 in clinical settings (Kopp & Gillberg, 2011) – the findings could potentially also be explained by lack of power, considering the generally low ASSQ scores among female study participants. While we are not aware of previous studies specifically focusing on sex differences in the association between vitamin D and ASD traits, a study by Schmidt et al. reported an inverse association between 25(OH)D levels and later ASD diagnosis exclusively among girls, while Windham et al. conversely demonstrated that girls who were vitamin D deficient at birth had lower odds of developing ASD (Schmidt, Niu, Eyles, Hansen, & Iosif, 2019; Windham et al., 2019). Importantly though, both of the aforementioned studies used 25(OH)D measurements collected at birth, whereas our analysis, specifically the latent profile analysis, reflects both the prenatal period and early childhood up until age 2 years. Furthermore, maternal vitamin D deficiency was rare in VIDI (3%; Hauta‐Alus et al., 2017) compared with Schmidt et al.'s (21%) and Windham et al.'s (13.7%) studies, potentially explaining the lack of observed associations between maternal 25(OH)D levels and ASD traits in both sexes.

The underlying causes of the lower 25(OH)D levels observed in children with ASD remain unknown. A shared genetic overlap between ASD and 25(OH)D has recently been suggested, based upon a large‐scale genome‐wide association study employing several methodologies, including bidirectional mendelian randomization in which no indications for a causal association were found (Yu et al., 2023). Additionally, previous studies have suggested that lifestyle factors, e.g., less time outdoors exposed to UV‐B radiation (Liu, Zhan, & Shao, 2015), and limited dietary habits (Esteban‐Figuerola, Canals, Fernández‐Cao, & Arija Val, 2019) may negatively impact vitamin D levels in children with ASD.

### Strengths and limitations

The study's strengths include a double‐blind randomized design on a well‐characterized population using standardized data collection. The study uses a prospective design and is, to our knowledge, the first to assess the associations between childhood vitamin D levels and ASD traits in a non‐clinical cohort setting. The outcome was assessed using ASSQ which has been validated for use in a general population setting, and for the Finnish population (Mattila et al., 2012). In Finland, the prevalence of ASD is 0.77% with an overrepresentation in boys to a factor of 3.3:1 (Delobel‐Ayoub et al., 2020). While not directly comparable, ASSQ scores above the suggested diagnostic cutoff point were only found among boys, in 1.4% (5/366) of the study participants. It has been suggested that ASSQ is less sensitive in identifying ASD traits that are more commonly seen in girls (Kopp & Gillberg, 2011), which may have contributed to the observed sex differences.

An important limitation was loss to follow‐up. The VIDI study originally comprised 987 children, 546 (55%) of whom took part in the VIDI2 follow‐up study. ASSQ results were available for 366 (67.0%) of these. Compared with those lost to follow‐up, children who remained in the study had more beneficial baseline characteristics, potentially limiting generalizability to more diverse populations. Attrition rates were, however, similar between supplementation groups and inverse probability weighting showed no indication of attrition bias. Maternal depressive symptoms were assessed using the CES‐D questionnaire administered in the immediate postpartum period, a time associated with an increased risk of mood volatility potentially limiting the generalizability of responses. Conversely, previous research indicates a high stability of maternal depressive symptoms, as assessed by CES‐D, from the prenatal period and up until the first year (Girchenko et al., 2022). Furthermore, by timing the CES‐D measurement to the immediate postnatal period, we minimize the risk that maternal depressive symptoms were influenced by the child's characteristics which might have been the case if we instead had used CES‐D measures from a later time point or at the time of the ASSQ assessments. VIDI2 took place during the SARS‐CoV‐2 pandemic and questionnaires were collected from September 2020 to May 2021. Although the pandemic may have influenced child mental health negatively (de Oliveira et al., 2022; Kauhanen et al., 2022), it is unlikely that this impact would differ between intervention groups. Dairy products and fat spreads have been fortified with vitamin D in Finland since 2003, improving population vitamin D status (Hauta‐Alus et al., 2017; Jääskeläinen et al., 2017). In the VIDI study, 95% of women used vitamin D supplementation during pregnancy with a daily supplemental vitamin D intake of 15.7 μg (range 0.0–197.5, interquartile range 10.0–20.0) (Hauta‐Alus et al., 2018). This could potentially limit generalizability in relation to studies performed in countries without systematic vitamin D fortification and lower population vitamin D levels. Furthermore, study findings may not be generalizable to children living at other geographical latitudes, and/or with ancestry other than Northern European.

## Conclusions

We found no indication that a higher‐than‐normal vitamin D supplementation during the first 2 years of life decreases the prevalence of ASD‐related traits. We did, however, observe an association between lower 25(OH)D levels at 1 and 2 years of age and across pregnancy up to 2 years and higher ASD‐related trait scores, albeit only in boys. Future studies are needed to examine potential underlying causes of this association. The lack of differences between intervention groups does not support conclusions about the causality of associations.

## Ethical considerations

Informed consent forms were collected from parents at recruitment and from parents and children at the 6–8‐year follow‐up. The original VIDI trial and follow‐up study (VIDI2) were approved by the ethics committee at the Hospital District of Helsinki and Uusimaa (ID 107/13/03/03/2012, HUS/1541/2019) on 2012‐03‐28 and 2019‐05‐15, respectively.

## Trial registration

ClinicalTrials.gov identifiers: NCT01723852 (VIDI), registered 2012‐11‐06 and available at https://www.clinicaltrials.gov/study/NCT01723852; NCT04302987 (VIDI2), registered 2020‐02‐24 and available at https://clinicaltrials.gov/study/NCT04302987.

**Key points**

**What's known?**

- Previous studies suggest an association between vitamin D and autism spectrum disorder (ASD) and related traits, but causality is unknown.

**What's new?**

- In this randomized clinical trial, we observed no differences in ASD‐related trait scores at ages 6–8 years between children who received the standard recommended daily dose of vitamin D3 supplementation (400‐IU) and those who received a higher‐than‐normal dosage (1200‐IU) between ages 2 weeks and 2 years.
- Lower pregnancy, 1‐, and 2‐year vitamin D levels were, however, associated with higher ASD trait scores, albeit only in boys.

**What's relevant?**

- Future studies are needed to examine potential underlying causes of the observed associations between vitamin D and ASD‐related traits, and the sex specificity of the found associations.

## Supporting information


**Appendix S1.** Supplementary methods.
**Table S1.** Attrition table for participants versus nonparticipants.
**Table S2.** Associations between covariates and autism spectrum screening questionnaire scores.
**Table S3.** Comparison of original and inverse probability weighting estimates.
**Table S4.** Association between seasonal variables and 25(OH)D concentration.
**Table S5.** Association between predictor variables and ASD symptoms at age 6–8 years in boys—Comparison between the current model and model with additional adjustment for internalizing problems.
**Table S6.** Association between predictor variables and ASD symptoms at age 6–8 years in girls—Comparison between the current model and model with additional adjustment for internalizing problems.
**Table S7.** Study participant characteristics by sex.
**Figure S1.** Latent profiles for 25(OH)D trajectories.

## Data Availability

Requests to access VIDI data can be sent to the corresponding author Samuel Sandboge (samuel.sandboge@tuni.fi). Requests to access individual‐level data are subject to assessment and approval by the VIDI study's management team to ensure the privacy of the participants, which is protected by law.
