# Supplementary material for: Vitamin D supplementation in the first 2 years and autism spectrum traits at 6–8 years – a randomized clinical trial
Source: J Child Psychol Psychiatry. 2026 Jan 5;67(7):1004–14. doi: 10.1111/jcpp.70110 (PMC13265620; doi:10.1111/jcpp.70110)
Supplement: Supplementary file 1 — Appendix S1. Supplementary methods. Table S1. Attrition table for participants versus nonparticipants. Table S2. Associations between covariates and autism spectrum screening questionnaire scores. Table S3. Comparison of original and inverse probability weighting estimates. Table S4. Association between seasonal variables and 25(OH)D concentration. Table S5. Association between predictor variables and ASD symptoms at age 6–8 years in boys—Comparison between the current model and model with additional adjustment for internalizing problems. Table S6. Association between predictor variables and ASD symptoms at age 6–8 years in girls—Comparison between the current model and model with additional adjustment for internalizing problems. Table S7. Study participant characteristics by sex. Figure S1. Latent profiles for 25(OH)D trajectories. [file JCPP-67-1004-s001.docx]

**Vitamin D supplementation in the first 2 years and autism spectrum traits at 6-8 years – A Randomized Clinical Trial**

**Supporting Information**

**Appendix S1.** Supplementary Methods

**Table S1.** Attrition Table for Participants vs Nonparticipants

**Table S2.** Associations Between Covariates and Autism Spectrum Screening Questionnaire scores

**Table S3 –** Comparison of original and Inverse probability weighting estimates

**Table S4.** Association between seasonal variables and 25(OH)D concentration

**Table S5 –** Association between predictor variables and ASD symptoms at age 6-8 years in boys – Comparison between current model and model with additional adjustment for internalizing problems

**Table S6 –** Association between predictor variables and ASD symptoms at age 6-8 years in girls – Comparison between current model and model with additional adjustment for internalizing problems

**Table S7.** Study Participant Characteristics by Sex

**Figure S1.** Latent profiles for 25(OH)D trajectories

This supplemental material has been provided by the authors to give readers additional information about their work.

**Appendix S1. Supplementary Methods**

**Inclusion and exclusion criteria**

The original inclusion and exclusion criteria have previously been described in detail.^1^ Exclusions from for the current study comprise 608 children who did not take part in the follow-up study, one child who was diagnosed with a rare genetic disorder after recruitment, as well as 12 children who were initially incorrectly including in the study but later were reclassified as filling the exclusion criteria. These 12 comprised 4 children who received intravenous antibiotics during the postpartum period, 3 children who were born small for gestational age, 2 children who were born large for gestational age, 2 children with ischemic stroke in the postpartum period, and one child with a genetic disorder.

**Attrition analysis**

In an attrition analysis (**Table S1**), we found that non-participants (n = 608) had mothers with lower pregnancy 25(OH)D concentrations, 32.3 (SD 7.7) vs 33.5 (SD 8.6) ng/mL who additionally smoked more often than the mothers of participants (19.9% vs 12.3%). The parents of non-participants were more likely to have lower attained education, and the average breastfeeding duration of non-participants was lower than among participants (10.3 (SD 5.6) vs 11.2 (SD 5.6) months). The attrition analysis did not include 13 children: 12 who did not fulfill the original inclusion criteria (6 from each supplementation group) and 1 child (from the 400 IU supplementation group) who was diagnosed with a rare genetic disorder after study recruitment.

The observed differences between participants and non-participants were similar between supplementation groups and the rate of attrition was similar.

**Baseline family characteristics and covariate creation**

Information on baseline health and lifestyle factors were obtained using self-administered questionnaires. Data on infant demographics, delivery, and gestation were obtained from electronic hospital records. The participants’ month of birth were categorized into four seasons: winter (December, January, February), spring (March, April, May), summer (June, July, August), and autumn (September, October, November). Parental education was classified into six categories, from 1 (comprehensive school) to 6 (university degree). These categories were used to create a dichotomous variable, low (less than a bachelor's degree), and high (bachelor's degree or above). Information on maternal smoking status was obtained from questionnaires and dichotomized. Missing education and smoking data were dummy coded. Study diaries provided information on breastfeeding duration. The Center for Epidemiological Studies Depression Scale (CES-D),^2^ was used to assess maternal depressive symptoms at birth. Mothers received the questionnaire at recruitment, i.e., after delivery. CES-D is a 20-item measure, each item is scored from 0 (rarely, none of the time) to 3 (most or almost all the time). A score of ≥16 indicates moderate depressive symptoms and a score of ≥24 indicates severe symptoms. In our material, scores were skewed and accordingly, they were square root transformed and normalized before being included in regression models. Mothers additionally completed the Beck Anxiety Inventory (BAI)^3^ at recruitment. BAI is a 21-item self-report measure of anxiety symptoms, with each item scored from 0 (not at all) to 3 (severely, I could barely stand it). Total scores range from 0 to 63, with 0–7 indicating minimal anxiety, 8–15 mild anxiety, 16–25 moderate anxiety, and 26–63 severe anxiety. Due to skewness, BAI scores were square root transformed and normalized before being included in the regression model testing for association with the outcome variable.

**Biochemical analyses**

25-hydroxyvitamin D (25(OH)D was analyzed using the IDS-iSYS fully automated immunoassay system with chemiluminescence detection (Immunodiagnostic Systems Ltd., Bolton, UK). The method has been found to have good linear agreement with liquid chromatography in tandem with mass spectroscopy (LC-MS, R^2^=0.942, in-house comparison of 67 samples). Mean (95% Confidence Interval (CI) value for the ratio of IDS-iSYS 25(OH)D to LC-MS 25(OH)D is 0.73 (0.68; 0.78) while intra-assay variations were 7%. Analysis was performed at the Pediatric Research Centre, University of Helsinki. Our laboratory participates in the inter-laboratory quality assessment scheme for vitamin D, DEQAS (Charing Cross Hospital, London, UK).

**Imputation**

Multiple imputation by fully conditional specification was used to impute 5 sets of data for 33 missing values for maternal depressive symptoms at birth, 1 missing value for breastfeeding duration, and one missing value for season of lab measurement at age 1 years. The imputation model included all variables originally considered as covariates, the outcome variable, the dichotomous supplementation group variable, as well as all 25(OH)D measurements variables.

**Sensitivity analysis**

To assess the potential impact of missing data, we re-ran all pooled analyses excluding individuals with missing data. The significance level, magnitude, and direction of all associations remained similar between the reduced and full sample.

**Inverse probability weighting analyses testing the impact of attrition**

We used inverse probability weighting (IPW) to assess whether findings might have been impacted by attrition bias – loss to follow-up (LTFU). The weight used was the inverse of the probability of LTFU. The Inverse probability was obtained using logistic regression, including all covariates used in the main analyses. To avoid extreme weights, the logistic regression predicted probabilities were truncated to the interval [0.01, 0.99]. So-called stabilized weights were used ensuring that the effective sample size (after weighting) would not change. IPW was computed, separately, for each imputed data set and the subsequent regression analyses in each imputed dataset were pooled accordingly. As in all main analyses, heteroskedasticity consistent covariance was used. Results are shown in **Table S3**. IPW estimates were of the same magnitude as those in the main analysis, suggesting that attrition bias would not explain the obtained results.

**Latent profile analysis**

in a post-hoc exploratory analysis, we employed latent profile analysis (LPA) to identify potential distinct trajectories of 25(OH)D levels from pregnancy through childhood up to age 2 years. Measurement data at all three time points, i.e. pregnancy, and ages 1, and 2 years, were available for 635 of the original study participants, 290 of whom are included in the current study. An á priori assumption of two separate trajectories, as anticipated based upon the two vitamin D_3_ supplementation groups, was supported by data analysis. When comparing the two chosen trajectories, one systematically had higher mean 25(OH)D concentrations at all measurement points (n = 316), compared to the other one (n=319) (**Figure S1**). Of those who were assigned to the high concentration trajectory, 67.0% (218/316) also belonged to the 1200 IU supplementation group. Conversely, of those who were assigned to low concentration trajectory, 79.3% (253/319) also belonged to the 400 IU supplementation group. The dichotomous trajectory variable was entered into regression models, replacing the supplementation group variable.

**Interaction**

To assess if the association vary by sex, interaction terms were calculated by multiplying each centered predictor variable (i.e., supplementation group status, 25(OH)D concentration measured during pregnancy, and at ages 1 and 2 years, and LPA group) with sex. These product terms were then entered alongside the related main predictor variable and sex into a series of linear regression models. Significant interaction was demonstrated between sex and 1- and 2-year 25(OH)D concentrations (p=.01 and p=.05, respectively) and hence, all analyses are also presented separately for boys and girls. *P* values for the remaining interaction analyses (intervention group * sex, latent profile * sex, and maternal 25(OH)D * sex) were .86, .06, and .91, respectively.

To explore whether observed associations vary by maternal pregnancy vitamin D status, interaction terms were created by multiplying supplementation group status, 25(OH)D at age 1 year, and 25(OH)D at age 2 years with maternal pregnancy 25(OH)D (all linear variables centered). These product terms were then entered into three separate linear regression models as a predictor variable together with the maternal 25(OH)D status variable plus the corresponding supplementation status group, 25(OH)D at age 1 year, and 25(OH)D at age 2 years, respectively. The outcome measure was the square root transformed, normalized ASSQ score variable. P-values for the supplementation group, 1-year vitamin D status, and 2-year vitamin D status interaction terms were 0.24, 0.18, and 0.16 for the three outcome variables, respectively, suggesting that the associations between supplementation group and childhood vitamin D levels do not vary according to maternal pregnancy vitamin D status.

**ASSQ scores and internalizing symptoms**

In a previous publication,^4^ we found that children in the 1200-IU vitamin D supplementation group had a lower risk of having clinically significant internalizing problems as assessed by the Child Behavior Checklist questionnaire (CBCL)^5^ compared with those in the 400-IU supplementation group (OR 0.40; 95% CI 0.17-0.94). Given that that symptoms and traits measured by ASSQ may overlap with those found in, e.g., internalizing problems, we conducted additional analyses.

We found a moderate correlation between ASSQ scores and CBCL internalizing score (0.40). In sex-stratified regression linear models, adding clinically significant internalizing symptoms (i.e., a T score of 64 or greater in the CBCL questionnaire) as an additional covariate, significance levels remained unchanged except in the case of the dichotomous LPA variable in boys which lost significance (**Tables S5** and **S6**).

These additional analyses should be interpreted with caution and given that the CBCL and ASSQ scores represent parallel and concurrently measured outcomes, internalizing problem scores should not be interpreted as a confounder of associations between early life vitamin D status and later ASD traits. On the contrary, internalizing problems can be an outcome of ASD, an early appearing neurodevelopmental disorder with strong genetic and prenatal aetiology, and/or other neurodevelopmental problems. In our previous study,^4^ we saw that vitamin D can predict internalizing problems, suggesting that internalizing problems might be an outcome of both ASD and early life vitamin D levels. Thus, in a causal framework, controlling for internalizing problems risks creating a collider bias.

**References**

1. Helve O, Viljakainen H, Holmlund-Suila E, et al. Towards evidence-based vitamin D supplementation in infants: vitamin D intervention in infants (VIDI) - study design and methods of a randomised controlled double-blinded intervention study. *BMC Pediatr*. 2017;17(1):91. doi:10.1186/s12887-017-0845-5

2. The CES-D Scale: A Self-Report Depression Scale for Research in the General Population - Lenore Sawyer Radloff, 1977. Accessed October 9, 2022. https://journals.sagepub.com/doi/abs/10.1177/014662167700100306

3. Beck AT, Epstein N, Brown G, Steer R. Beck Anxiety Inventory. Published online January 9, 2012. doi:10.1037/t02025-000

4. Sandboge S, Räikkönen K, Lahti-Pulkkinen M, et al. Effect of Vitamin D3 Supplementation in the First 2 Years of Life on Psychiatric Symptoms at Ages 6 to 8 Years: A Randomized Clinical Trial. *JAMA Network Open*. 2023;6(5):e2314319. doi:10.1001/jamanetworkopen.2023.14319

5. Achenbach T. Manual for the Child Behaviour Check-List/4-18 and 1991 Profile. Accessed November 18, 2025. https://cir.nii.ac.jp/crid/1370004237624813324

**Table S1 – Attrition table for Participants vs Nonparticipants**

| **Characteristic** | **Nonparticipants (n = 608)** | | **Participants (n = 366)** | | **MD (95% CI)** | ***P* value** |
| --- | --- | --- | --- | --- | --- | --- |
| **Child** |  |  |  |  |  |  |
| Female sex, No. (%) | 309 (50.8) | 608 | 176 (48.1) | 366 |  | .41 |
| Gestational length, mean (SD), d | 281.6 (7.7) | 608 | 280.8 (7.5) | 366 | -0.8 (-1.8; 0.2) | .12 |
| Season of birth |  | 608 |  | 366 |  | .01 |
| Winter, No. (%) | 125 (20.6) |  |  | 64 (17.5) |  |  |
| Spring, No. (%) | 268 (44.1) |  |  | 132 (36.1) |  |  |
| Summer, No. (%) | 124 (20.5) |  |  | 93 (25.4) |  |  |
| Autumn, No. (%) | 91 (15.0) |  |  | 77 (21.0) |  |  |
| Length of breastfeeding, mean (SD), m | 10.3 (5.6) | 488 | 11.2 (5.6) | 365 | 0.9 (0.1; 1.6) | .03 |
| Belonging to 1200 IU group, No (%) | 235 (49.1) | 479 | 189 (51.6) | 366 |  | .46 |
| **Mother** |  |  |  |  |  |  |
| Age at delivery, mean (SD), y | 30.9 (4.6) | 516 | 31.5 (4.1) | 363 | 0.6 (-0.02; 1.1) | .06 |
| Smoking at childbirth (yes), No. (%) | 103 (19.9) | 518 | 45 (12.3) | 365 |  | .003 |
| Pregnancy 25(OH)D concentration |  |  |  |  |  |  |
| Mean (SD), ng/mL | 32.3 (7.7) | 500 | 33.5 (8.6) | 308 | 1.2 (0.03; 2.4) | .04 |
| < 30 ng/mL, No. (%) | 299 (58.8) | 500 | 206 (66.9) | 308 |  | .04 |
| Educational level (high), No. (%) | 309 (71.2) | 434 | 255 (83.1) | 307 |  | <.001 |
| Depressive symptoms at childbirth, median (IQR), score^a^ | 11 (7–15) | 441 | 11 (8–15) | 333 |  | .97 |
| CES-D score ≥ 16, No. (%)^b^ | 108 (24.5) | 441 | 77 (23.1) | 333 |  | .66 |
| Anxiety symptoms at childbirth, BAI score^c^ | 5 (2-8) | 449 | 5 (2-8) | 330 |  | .91 |
| BAI score categorized^d^ |  |  |  |  |  | .88 |
| BAI score 0-7, No. (%) | 319 (71.0) | 449 | 235 (71.2) | 330 |  |  |
| BAI score 8-15, No. (%) | 113 (25.2) | 449 | 82 (24.8) | 330 |  |  |
| BAI score 16-25, No. (%) | 14 (3.1) | 449 | 12 (3.6) | 330 |  |  |
| BAI score ≥ 26, No. (%) | 3 (0.7) | 449 | 1 (0.3) | 330 |  |  |
| **Father** |  |  |  |  |  |  |
| Educational level (high), No. (%) | 255 (59.6) | 428 | 207 (68.8) | 301 |  | .01 |

Abbreviations: IU, International units; SD, standard deviations; d, days, m, months; y, years; 25(OH)D, serum 25 hydroxyvitamin D; CES-D, Center for Epidemiological Studies Depression Scale

SI conversion factor: To convert 25(OH)D to nmol/L, multiply by 2.496.

^a^Depressive symptoms assessed using the Center for Epidemiological Studies Depression Scale.

^b^Scores of 16 or above indicate a risk of clinical depression.
^c^Symptoms of anxiety were measured using Beck Anxiety Inventory, square root transformed due to skewness and converted to Z-scores (0 = mean, 1 = 1 SD).

^d^Total BAI scores range from 0 to 63, with 0–7 indicating minimal anxiety, 8–15 mild anxiety, 16–25 moderate anxiety, and 26–63 severe anxiety

**Table S2 – Association between covariates and ASSQ scores^a^**

| **Characteristic** | **MD** | **(95% CI)** | ***P* value** |
| --- | --- | --- | --- |
| **Child** |  |  |  |
| Sex, female vs male (ref) | -0.45 | -0.65; -0.25 | <.001 |
| Gestational length, d | -0.01 | -0.02; 0.003 | .14 |
| Season of birth, winter (ref) |  |  |  |
| Spring | -0.25 | -0.54; 0.04 | .09 |
| Summer | -0.03 | -0.36; 0.31 | .88 |
| Autumn | -0.13 | -0.49; 0.23 | .49 |
| **At 12-month follow-up** |  |  |  |
| Length of breastfeeding, m | -0.02 | -0.04; -0.01 | .02 |
| **At 6–8-year follow-up** |  |  |  |
| Age, y | 0.10 | -0.13; 0.34 | .39 |
| **Mother** |  |  |  |
| Age at delivery, y | -0.01 | -0.04; 0.01 | .39 |
| Smoking at childbirth yes vs no (ref) | -0.12 | -0.45; 0.21 | .47 |
| Depressive symptoms at childbirth, CES-D score^b^ | 0.14 | 0.04; 0.25 | .01 |
| Anxiety symptoms at childbirth, BAI score^c^ | 0.09 | -0.03; 0.21 | .15 |
| Educational level low vs high (ref) | 0.37 | 0.07; 0.67. | .01 |
| **Father** |  |  |  |
| Educational level, low vs high (ref) | 0.26 | 0.02; 0.50 | .04 |

Abbreviations: ASSQ, Autism Spectrum Screening Questionnaire; MD, mean difference; d, days; m, months; y, years; 25(OH)D, serum 25 hydroxyvitamin D; CES-D, Center for Epidemiological Studies Scale.

^a^ASSQ raw scores square root transformed due to skewness and converted to Z-scores (0 = mean, 1 = 1 SD).

^b^Depressive symptoms were measured using the Center for Epidemiological Studies Scale, square root transformed due to skewness and converted to Z-scores (0 = mean, 1 = 1 SD).

^c^Symptoms of anxiety were measured using Beck Anxiety Inventory, square root transformed due to skewness and converted to Z-scores (0 = mean, 1 = 1 SD).

**Table S3 – Comparison of original and Inverse probability weighting estimates**

|  | **Original analysis** | |  | **IPW estimates** | |
| --- | --- | --- | --- | --- | --- |
|  | B (95% CI) | *P* value |  | B (95% CI) | *P* value |
| **Supplementation group, 1200-IU vs 400-IU** |  |  |  |  |  |
| Crude | -0.002 (-0.21; 0.20) | .98 |  | 0.02 (-0.20; 0.23) | .89 |
| Adjusted | -0.002 (-0.20; 0.20) | .98 |  | 0.02 (-0.19; 0.22) | .87 |
| **25(OH)D trajectory group, high vs low** |  |  |  |  |  |
| Crude | -0.24 (-0.46; -0.01) | .04 |  | -0.23 (-0.47; 0.002) | .05 |
| Adjusted | -0.22 (-0.44; -0.004) | .05 |  | -0.23 (-0.45; -0.01) | .04 |
| **Maternal 25(OH)D levels during pregnancy** |  |  |  |  |  |
| Crude | -0.01 (-0.02; 0.01) | .31 |  | -0.01(-0.02; 0.005) | .23 |
| Adjusted | -0.004 (-0.02; 0.01) | .54 |  | -0.01 (-0.02; 0.01) | .44 |
| **Child’ s 1-year 25(OH)D levels** |  |  |  |  |  |
| Crude | -0.01 (-0.02; 0.002) | .14 |  | -0.01 (-0.02; 0.002) | .14 |
| Adjusted | -0.01 (-0.02; 0.003) | .16 |  | -0.01 (-0.02; 0.002) | .14 |
| **Child’s 2-year 25(OH)D levels** |  |  |  |  |  |
| Crude | -0.01 (-0.02; 0.001) | .09 |  | -0.01 (-0.02; 0.0005) | .06 |
| Adjusted | -0.01 (-0.02; 0.002) | .12 |  | -0.01 (-0.02; 0.001) | .10 |

**Table S4 – Association between seasonal variables and 25(OH)D concentration at three time points**

|  | **maternal 25(OH)D** | | | **1-year 25(OH)D** | | | **2-year 25(OH)D** | | |
| --- | --- | --- | --- | --- | --- | --- | --- | --- | --- |
| **Characteristic** | **MD** | **(95% CI)** | ***P* value** | **MD** | **(95% CI)** | ***P* value** | **MD** | **(95% CI)** | ***P* value** |
| Season of birth, winter (ref) |  |  |  |  |  |  |  |  |  |
| Spring | 1.3 | -1.7 to 4.2 | .87 | -3.8 | -7.6; 0.1 | .05 | -1,9 | -5.2; 1.3 | .24 |
| Summer | 2.5 | -0.3 to 5.3 | .08 | -4.1 | -8.3; 0.1 | .05 | 1.6 | -1.9; 5.1 | .36 |
| Autumn | 2.5 | -0.05 to 5.1 | .05 | -1.0 | -5.1; 3.2 | .65 | 2.8 | -0.8; 6.5 | .13 |

Abbreviations: 25(OH)D, serum 25 hydroxyvitamin D; MD, mean difference, CI, confidence interval

**Table S5 – Association between predictor variables and ASD symptoms at age 6-8 years in boys – Comparison between current model and model with additional adjustment for internalizing problems**

|  | **Current model** | | **Additional adjustment for internalizing problems** | |
| --- | --- | --- | --- | --- |
|  | B (95% CI) | *P* | B (95% CI) | *P* |
| **Supplementation group, 1200-IU vs 400-IU** | 0.02 (-0.28; 0.32) | .90 | 0.06 (-0.25; 0.36) | .72 |
| **25(OH)D trajectory group, high vs low** | -0.45 (-0.80; -0.10) | .01 | -0.33 (-0.69; 0.03) | .08 |
| **Maternal 25(OH)D levels** | -0.004 (-0.02; 0.01) | .68 | -0.004 (-0.02; 0.01) | .61 |
| **Child’ s 1-year 25(OH)D** | -0.02 (-0.03; -0.01) | .003 | -0.02 (-0.03; -0.004) | .01 |
| **Child’s 2-year 25(OH)D** | -0.02 (-0.03; -0.005) | .01 | -0.01 (-0.03; -0.001) | .04 |

**Table S6 – Association between predictor variables and ASD symptoms at age 6-8 years in girls – Comparison between current model and model with additional adjustment for internalizing problems**

|  | **Current model** | | **Additional adjustment for internalizing problems** | |
| --- | --- | --- | --- | --- |
|  | B (95% CI) | *P* | B (95% CI) | *P* |
| **Supplementation group, 1200-IU vs 400-IU** | -0.01 (-0.27; 0.25) | .95 | -0.03 (-0.24; 0.30) | .84 |
| **25(OH)D trajectory group, high vs low** | 0.004 (-0.27; 0.28) | .98 | 0.14 (-0.15; 0.43) | .33 |
| **Maternal 25(OH)D levels** | -0.01 (-0.02; 0.01) | .50 | -0.004 (-0.02; 0.02) | .71 |
| **Child’ s 1-year 25(OH)D** | 0.01 (-0.01; 0.02) | .42 | 0.09 (-0.004; 0.02) | .19 |
| **Child’s 2-year 25(OH)D** | 0.004 (-0.01; 0.02) | .53 | 0.01 -0.01; 0.02) | .43 |

**Table S7 – Study participant characteristics by sex**

| **Characteristic** | **Female (n = 176)** | | **Male (n = 190)** | | **MD (95% CI)** | ***P* value** |
| --- | --- | --- | --- | --- | --- | --- |
| **Child** |  |  |  |  |  |  |
| Gestational length, mean (SD), d | 281.6 (7.3) | 176 | 280.1 (7.7) | 190 | -1.5 (-3.1; -0.002) | .05 |
| Season of birth |  | 176 |  | 190 |  | .79 |
| Winter, No. (%) | 30 (17.0) |  | 34 (17.9) |  |  |  |
| Spring, No. (%) | 67 (38.1) |  | 65 (34.2) |  |  |  |
| Summer, No. (%) | 41 (23.3) |  | 52 (27.4) |  |  |  |
| Autumn, No. (%) | 38 (21.6) |  | 39 (20.5) |  |  |  |
| **Mother** |  |  |  |  |  |  |
| Age at delivery, mean (SD), y | 31.5 (4.2) | 175 | 31.5 (4.1) | 188 | -0.002 (-0.86; 0.85) | .995 |
| Smoking at childbirth (yes), No. (%) | 18 | 175 | 27 | 190 |  |  |
| Pregnancy 25(OH)D concentration |  |  |  |  |  |  |
| Mean (SD), ng/mL | 33.3 (7.6) | 149 | 33.8 (9.5) | 159 | 0.5 (-1.4; 2.5) | .59 |
| < 30 ng/mL, No. (%) | 103 (69.1) | 149 | 103 (64.8) | 159 |  | .42 |
| Educational level (high), No. (%) | 129 (87.2) | 148 | 126 (79.2) | 159 |  | .07 |
| Depressive symptoms at childbirth, median (IQR), score^a^ | 11 (7.8–14) | 158 | 10 (8.0–16.0) | 175 |  | .98 |
| CES-D score ≥ 16 No. (%)^b^ | 33 (20.9) | 158 | 44 (25.1) | 175 |  | .36 |
| **Father** |  |  |  |  |  |  |
| Educational level (high), No. (%) | 105 (71.9) | 146 | 102 (65.8) | 155 |  | .25 |
| **Child** |  |  |  |  |  |  |
| **At 12-month follow-up** |  |  |  |  |  |  |
| Length of breastfeeding, mean (SD), months | 11.5 (5.5) | 175 | 10.9 (5.7) | 190 | -0.6 (-1.8; 0.6) | .31 |
| 25(OH)D concentration |  |  |  |  |  |  |
| Mean (SD), ng/mL | 41.5 (12.5) | 167 | 40.0 (11.5) | 176 | -1.5 (-4.1; 1.1) | .25 |
| < 30 ng/mL, No. (%) | 28 (16.8) | 167 | 35 (20.0) | 176 |  | .46 |
| **At 24-month follow-up** |  |  |  |  |  |  |
| 25(OH)D concentration |  |  |  |  |  |  |
| Mean (SD), ng/mL | 42.8 (10.8) | 175 | 41.0 (11.3) | 188 | -1.8 (-4.1; 0.5) | .12 |
| < 30 ng/mL, No. (%) | 22 (12.6) | 175 | 34 (18.1) | 188 |  | .15 |
| **At 6–8-year follow-up** |  |  |  |  |  |  |
| Age, mean (SD), y | 7.1 (0.4) | 176 | 7.2 (0.4) | 190 | 0.04 (-0.05; 0.1) | .43 |
| ASSQ score, median (IQR) | 1.5 (0.0;3.0) | 176 | 3.0 (1.0; 6.0) |  |  | <.001 |
| **Belonging to low 25(OH)D trajectory group, No. (%)** | 91 (51.7) | 176 | 98 (49.5) | 190 |  | .98 |

Abbreviations: IU, International units; SD, standard deviations; d, days, m, months; y, years; 25(OH)D, serum 25 hydroxyvitamin D; CES-D, Center for Epidemiological Studies Depression Scale

SI conversion factor: To convert 25(OH)D to nmol/L, multiply by 2.496.

^a^Depressive symptoms assessed using the Center for Epidemiological Studies Depression Scale. ^b^Scores of 16 or above indicate a risk of clinical depression.


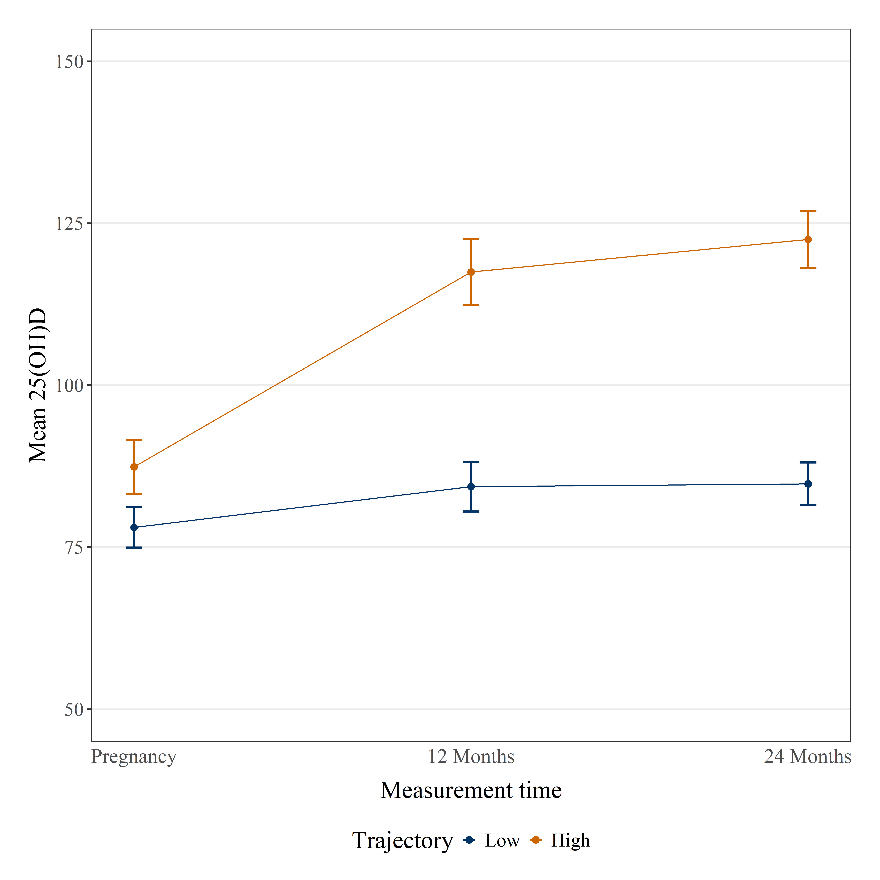


**Figure S1**. Latent profiles for 25(OH)D trajectories, based upon 635 serum measurements, (n=316 in the orange curve; n=319 in the blue curve).
